# Contribution of Maternal Antiretroviral Therapy and Breastfeeding to 24-Month Survival in Human Immunodeficiency Virus-Exposed Uninfected Children: An Individual Pooled Analysis of African and Asian Studies

**DOI:** 10.1093/cid/cix1102

**Published:** 2017-12-21

**Authors:** Shino Arikawa, Nigel Rollins, Gonzague Jourdain, Jean Humphrey, Athena P Kourtis, Irving Hoffman, Max Essex, Tim Farley, Hoosen M Coovadia, Glenda Gray, Louise Kuhn, Roger Shapiro, Valériane Leroy, Robert C Bollinger, Carolyne Onyango-Makumbi, Shahin Lockman, Carina Marquez, Tanya Doherty, François Dabis, Laurent Mandelbrot, Sophie Le Coeur, Matthieu Rolland, Pierre Joly, Marie-Louise Newell, Renaud Becquet

**Affiliations:** 1 University of Bordeaux, Inserm, Bordeaux Population Health Research Center, Team IDLIC, France; 2 Department of Maternal, Newborn, Child and Adolescent Health, World Health Organization, Geneva, Switzerland; 3 Institut de recherche pour le développement UMI 174-PHPT, Marseille, France; 4 Faculty of Associated Medical Sciences, Chiang Mai University, Thailand; 5 Department of Immunology and Infectious Diseases, Harvard T.H. Chan School of Public Health, Boston, Massachusetts; 6 Department of International Health, Center for Global Health, Bloomberg School of Public Health, Johns Hopkins University, Baltimore, Maryland; 7 Women’s Health and Fertility Branch, Division of Reproductive Health, National Center for Chronic Disease Prevention and Health Promotion, Centers for Disease Control and Prevention; 8 Emory University School of Medicine and Eastern Virginia Medical School, Atlanta, Georgia; 9 Division of Infectious Diseases, Department of Medicine, University of North Carolina School of Medicine, Chapel Hill; 10 Sigma3 Services SÀRL, Nyon, Switzerland; 11 Maternal Adolescent and Child Health, University of the Witwatersrand, Johannesburg; 12 South African Medical Research Council, Cape Town; 13 Perinatal HIV Research Unit, University of the Witwatersrand, Johannesburg, South Africa; 14 Gertrude H. Sergievsky Center, College of Physicians and Surgeons, and Department of Epidemiology, Mailman School of Public Health, Columbia University, New York, New York; 15 Inserm, Centre de recherche Inserm U1027, Université Paul Sabatier Toulouse 3, France; 16 Center for Clinical Global Health Education, Johns Hopkins University, Baltimore, Maryland; 17 Makerere University–Johns Hopkins University Research Collaboration/MU-JHU CARE LTD, Kampala, Uganda; 18 Division of HIV, Infectious Diseases and Global Medicine, University of California San Francisco, and Zuckerberg San Francisco General Hospital; 19 University Paris-Diderot, Assitance Publique Hôpitaux de Paris; 20 Institut National d’Etudes Démographiques (Ined), Paris; 21 University of Bordeaux, Inserm, Bordeaux Population Health Research Center, Team Biostatistics, France; 22 Institute for Developmental Science and Global Health Research Institute, Faculty of Medicine, University of Southampton, United Kingdom

**Keywords:** HIV-exposed uninfected, children, infants, mortality, Asia, Africa

## Abstract

**Background:**

Human immunodeficiency virus (HIV)–infected pregnant women increasingly receive antiretroviral therapy (ART) to prevent mother-to-child transmission (PMTCT). Studies suggest HIV-exposed uninfected (HEU) children face higher mortality than HIV-unexposed children, but most evidence relates to the pre-ART era, breastfeeding of limited duration, and considerable maternal mortality. Maternal ART and prolonged breastfeeding while on ART may improve survival, although this has not been reliably quantified.

**Methods:**

Individual data on 19 219 HEU children from 21 PMTCT trials/cohorts undertaken from 1995 to 2015 in Africa and Asia were pooled to estimate the association between 24-month mortality and maternal/infant factors, using random-effects Cox proportional hazards models. Adjusted attributable fractions of risks computed using the predict function in the R package “frailtypack” were used to estimate the relative contribution of risk factors to overall mortality.

**Results:**

Cumulative incidence of death was 5.5% (95% confidence interval, 5.1–5.9) by age 24 months. Low birth weight (LBW <2500 g, adjusted hazard ratio (aHR, 2.9), no breastfeeding (aHR, 2.5), and maternal death (aHR, 11.1) were significantly associated with increased mortality. Maternal ART (aHR, 0.5) was significantly associated with lower mortality. At the population level, LBW accounted for 16.2% of 24-month mortality, never breastfeeding for 10.8%, mother not receiving ART for 45.6%, and maternal death for 4.3%; combined, these factors explained 63.6% of deaths by age 24 months.

**Conclusions:**

Survival of HEU children could be substantially improved if public health practices provided all HIV-infected mothers with ART and supported optimal infant feeding and care for LBW neonates.

## Teaser

Pooled results from 21 studies involving more than 19 000 human immunodeficiency virus–exposed but uninfected children show that an estimated two-thirds of infant deaths are attributable to lack of antiretroviral treatment for mothers, low birth weight, never being breastfed, and mother’s death.

Antiretroviral therapy (ART) to prevent mother-to-child transmission (PMTCT) of human immunodeficiency virus (HIV) has dramatically reduced the number of HIV-infected infants [1, 2]. As more women living with HIV survive and become pregnant, the number of HIV-exposed uninfected (HEU) children continues to increase [3]. Several studies have shown that HEU children may experience worse health outcomes than HIV-unexposed uninfected (HUU) children in the same setting [4–10], although this has not been confirmed elsewhere [11–14]. Most evidence relates to the era before widespread use of ART for PMTCT and for treatment, when increased mortality in HEU children was associated with poor maternal health and lack of prolonged breastfeeding. Maternal ART for life and prolonged breastfeeding with the protection of ART could ameliorate such negative associations [15, 16], but this has not yet been reliably quantified.

By pooling available individual data on HEU children from clinical trials and observational studies, from both the pre- and post-ART era, we assessed mortality risk in HEU children in Africa and Asia and associated factors. We also estimated the relative importance of identified risk factors in mediating poor outcomes among HEU children.

## METHODS

In a recent systematic review [17], we electronically searched 2 bibliographic databases, PubMed and Scopus, for articles published from 2004 to 2015 using the following keywords: HIV, Mortality, and Child or Infant, without restrictions on type or region of study, limited to English and French. Titles and abstracts were assessed; retained articles were subject to full-text reviews with identification of additional references. Additionally, we identified PMTCT trials with potential data on mortality in HEU children. A total of 29 studies were identified, and their principal investigators were contacted. One declined participation [18], 5 were unable to share data [5, 19–22] and 2 did not meet the inclusion criteria [23, 24], leaving 21 studies for the pooled 24-month mortality analysis: 16 from sub-Saharan Africa [4, 8, 25–38] and 5 from Asia [39–42]. Of these, 17 were randomized trials and 4 were observational studies conducted at different times (Supplementary Table S1), with varying sample sizes and follow-up durations (Table 1).

**Table 1.** Characteristics of the Mothers and Children in Included in the Studies/Trials (N = 19219)

Trial/Study	No. of Children	Follow-up Duration (days)			Birth Weight (<2500 g)		Ever Breastfed		Breastfeeding Duration (days)			Antenatal Maternal CD4 (cells/mm^3^)			Maternal Death		Child Death		Child Age at Death (days)		
	n	Median	Q1	Q3	n	%	n	%	Median	Q1	Q3	Median	Q1	Q3	n	%	n	%	Median	Q1	Q3
**BAN**	2250	336	254	338	174	7.7	2250	100	169	167	196	439	330	582	9	0.4	56	2.5	190	61	283
**Ditrame(ANRSa**)	312	551	385	641	43	13.8	306	98.1	283	188	505	596	403	781	18	4.5	32	10.3	77	4	130
**Ditrame(ANRSb**)	90	546	284	604	15	16.7	89	98.9	342	237	484	576	420	809	9	8.7	16	17.8	71	36	176
**Ditrame Plus**	688	730	538	734	83	12.1	395	57.4	124	96	200	411	258	569	13	1.7	53	7.7	2	1	79
**HIVIGLOB/SWEN Uganda**	575	546	541	549	62	10.8	574	99.9	105	77	150	436	292	596	2	0.3	21	3.7	70	15	207
**HIVNET024**	1457	377	340	401	139	9.5	1457	100	284	108	366	366	240	523	40	2.4	67	4.6	173	116	273
**Good Start**	259	252	252	252	31	12.0	259	52.8	252	252	252	.	.	.	13	2.7	8	3.1	74	56	123
**Kesho Bora**	965	561	543	730	90	9.3	723	74.9	156	80	192	341	258	435	10	1.0	66	6.8	126	29	213
**Mashi**	1102	1096	730	1461	80	7.3	533	48.3	176	119	182	370	244	517	31	2.8	83	7.5	75	12	224
**Mma Bana**	702	731	730	733	102	14.5	680	96.9	178	141	181	347	231	484	11	1.6	35	5.0	151	6	243
**PEP**	779	210	153	246	128	16.4	405	52.0	81	38	123	477	323	664	11	1.4	18	2.3	109	71	138
**PHPT-1**	1283	551	546	558	126	9.8	0	0	.	.	.	362	240	510	49	3.8	3	0.2	185	182	548
**PHPT-2**	1805	368	365	373	172	9.5	0	0	.	.	.	376	247	531	6	0.3	4	0.2	283	180	289
**PHPT-5 1st**	407	731	553	737	51	12.5	0	0	.	.	.	454	366	569	0	0	0	0	.	.	.
**PHPT-5 2nd**	310	189	184	207	62	20.0	0	0	.	.	.	361	250	489	0	0	1	0.3	156	156	156
**PROMOTE2**	361	404	310	408	69	19.1	357	98.9	365	280	392	377	280	506	9	2.5	9	2.5	138	22	238
**SWEN**	628	366	364	368	177	28.2	627	99.8	101	98	182	472	324	667	8	1.3	14	2.2	46	27	188
**Tshipidi**	429	735	731	774	60	14.0	34	7.93	181	44	184	426	320	577	7	1.6	22	5.1	23	2	166
**VTS**	936	703	485	779	101	10.8	854	91.2	224	177	281	479	342	642	44	4.7	39	4.2	125	54	261
**ZEBS**	763	729	368	730	82	10.8	763	100	182	126	487	361	237	498	47	6.2	93	12.2	245	129	394
**Zvitambo**	3118	464	365	729	474	15.2	3113	99.8	456	365	553	423	279	593	134	4.3	245	7.9	82	38	201

Maternal antiretroviral exposure was categorized as none; single/double peripartum antiretrovirals for PMTCT; 3-drug ART for PMTCT given antenatally and postnatally until cessation of breastfeeding when breastfeeding or until delivery when exclusively formula-fed; or 3-drug ART for life, prescribed beyond breastfeeding cessation per World Health Organization (WHO) HIV treatment and prevention recommendations [43, 44]. Mothers with missing information on antiretroviral use (n = 44) were assumed to have followed the relevant study protocol [28, 35] and thus categorized into the single/double antiretroviral PMTCT. The final HIV status of each child was defined by study-specific criteria. In our analyses, each child contributed from birth to 24 months of age, with right-censoring in case of death, end of study follow-up, and loss to follow-up. We restricted analyses to HEU children with information on breastfeeding and excluded 457 children with unknown infant feeding status. Mortality rates per 100 child-years of follow-up were estimated by maternal and child characteristics. We used the Kaplan-Meier method to estimate survival curves and the log-rank test to test for differences between groups.

Associations between 24-month mortality and the following factors were assessed: residence (rural vs urban/peri-urban), sex, low birth weight (LBW; <2500 g), breastfeeding (ever/never), maternal education (none/primary vs above), maternal age at delivery (5-year categories), maternal antiretroviral exposure (fixed), and maternal vital status (time-dependent). Children known to have initiated breastfeeding but with unknown weaning date (n = 1032) were considered to have been breastfed from birth to either age 6 months per WHO feeding guidance at the time [45], study exit date, or date of mother’s death, whichever occurred first. We used random-effects Cox proportional hazards models to estimate the association between 24-month mortality and potential risk factors, accounting for heterogeneity between studies. The final multivariable model included region (Africa vs Asia) as a fixed effect and adjusted for maternal antenatal CD4 cell count (categorical) because CD4 counts and ART eligibility varied widely between studies. Data from different sites in Kesho Bora [31] and HIVNET024 [30] were treated separately. Missing data were included as a separate category to maintain sample size. We used a stepwise-descending approach for selection of variables in multivariable models, which included variables that were statistically significant in univariate analyses (at a *P* value < .1, except for maternal antiretroviral exposure, which was maintained in the model independent of statistical significance). In the final model, statistical significance was reached when the *P* value was < .05. We also analyzed the association between weaning and survival among breastfed children only (n = 13418), with breastfeeding cessation defined in a time-varying manner.

We assessed the combined effects of breastfeeding and maternal 3-drug ART (for PMTCT or for life) on mortality, classifying observation time for each HEU child into 4 categories defined by child being breastfed (yes/no) and mother being on 3-drug ART (yes/no), with breastfeeding and ART variables being time dependent. When the date of ART end was unknown, ART was assumed to have continued until the weaning date or 6 months post-partum [45], whichever came first. The association between 24-month mortality and breastfeeding/maternal 3-drug ART was assessed in multivariable analyses using Cox proportional hazards models and allowing for heterogeneity between studies/trials and adjusting for region as fixed effect and maternal antenatal CD4 cell count and birth weight (<2500 g) as categorical variables.

Finally, to investigate the relative contribution of risk factors to overall 24-month mortality in HEU children, we estimated the adjusted attributable fractions (aAFs) of risks based on our final multivariable model [46, 47]. The AF for a given factor was the number of deaths attributable to the factor divided by the total number of deaths in our population if the prevalence of other factors remained at the same level. To do this, we first obtained the total number of deaths at a given time by summing the individual predicted probabilities of survival for each child based on the predict function in the R package “frailtypack” [48], then we subtracted this number from the total population to derive the number of deaths. To estimate the number of deaths attributable to the exposure of interest, we computed the number of deaths in the population as if it was not exposed to the factor while exposures to other risk factors were unchanged. Nonexposure was simulated by setting all children to the reference category. For example, for deaths associated with LBW, all children were classified into the category of having birth weight great than 2500 g. The number of deaths attributable to a specific factor was the difference between the total number of deaths calculated previously and the number of deaths in the unexposed population. We estimated the aAFs of the identified risk factors at 6, 12, and 24 months of age and computed 95% confidence intervals (CIs) using bootstrapping [49]. All statistical analyses were performed using SAS version 9.3 (SAS Institute, Cary, North Carolina). For the estimates of AF, we used the R package’s “frailtypack” [48] and “boot” [49] using R version 3.3.2 (R Development Core Team, 2004).

## RESULTS

**Figure 1.**
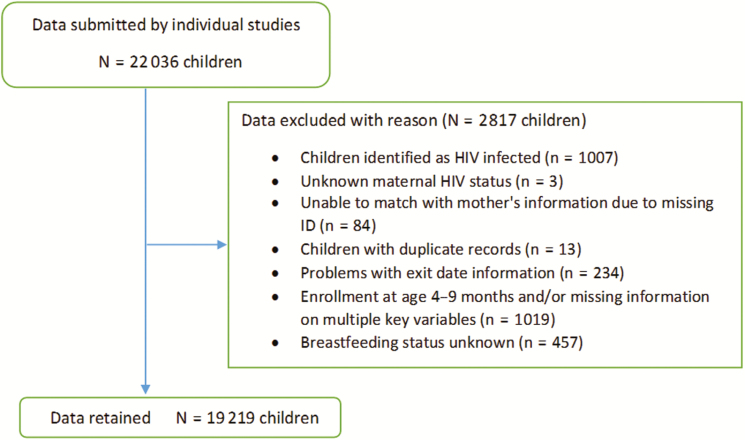
Flow chart of the children included in the pooled analyses. Abbreviations: HIV, human immunodeficiency virus; ID, identification.

A total of 19219 HEU children were part of the analyses (Figure 1). Maternal/child baseline characteristics are shown in Table 2. Median child follow-up was 404 days (interquartile range [IQR], 336–712). More than 75% of children were born in sub-Saharan Africa, mostly Southern Africa; nearly 70% were born prior to 2005. Most children were ever breastfed (69.8%) for a median 181 days (IQR, 126–365). Maternal antiretroviral exposure varied across studies (Supplementary Table S2), reflecting the timing of the study and prevailing ART and PMTCT recommendations [44]. Overall, 23% of mothers received no antiretrovirals, 61% received mono/dual peripartum antiretrovirals for PMTCT, 12% received 3-drug ART for PMTCT, and only 4% were on ART for life. Median antenatal CD4 count was 405 cells/mm^3^ (IQR, 280–563); 58% of mothers had a CD4 count >350 cells/mm^3^ at the first antenatal visit. Median antenatal CD4 count in women who received ART for life was low at 214 cells/mm^3^ (IQR, 147–361). Median duration of ART was 178 (IQR, 152–196) and 443 (IQR, 371–730) days for 3-drug ART for PMTCT and ART for life, respectively. Information on maternal viral load was missing for 16%; among those with available information, median antenatal viral load was 4.0 log_10_ copies/mL (IQR, 3.3–4.6).

**Table 2.** Baseline Characteristics of the Study Population (N = 19219)

Child Characteristics		
**Follow-up duration (days) (median, IQR**)		
	404	336–712
**Region (N, %**)		
East Africa	1620	8.4
Southern Africa	11769	61.2
West Africa	1397	7.3
South Asia	628	3.3
Southeast Asia	3805	19.8
**Residence (N, %**)		
Rural	1868	9.8
Urban/Peri-urban	17351	90.2
**Access to piped water at home (N, %**)		
Yes	6182	32.2
No	4268	22.2
Unknown	8769	45.6
**Years of birth (N, %**)^**a**^		
1995–1999	4659	24.2
2000–2004	8419	43.8
2005–2009	4852	25.3
2010–2014	1289	6.7
**Sex (N, %**)		
Male	9839	51.2
Female	9376	48.8
Unknown	4	0.0
**Birth weight (N, %**)		
≥2500 g	16653	86.6
<2500 g	2321	12.1
Unknown	245	1.3
**Child ever breastfed (N, %**)		
Yes	13418	69.8
No	5801	30.2
**Breastfeeding duration (days) (median, IQR) (N** = **13418**)		
	181	126–365
**Maternal characteristics**		
**Education (N, %**)		
None/Primary	8676	45.1
Secondary or above	9688	50.4
Unknown	855	4.5
**Maternal age at delivery (years) (N, %**)		
15–20	2074	10.8
21–25	6159	32.1
26–30	5175	26.9
31–35	2545	13.2
>35	1011	5.3
Unknown	2255	11.7
**Maternal ARVs/ART (N, %**)		
No ARVs	4392	22.8
Single or double ARVs for PMTCT	11805	61.4
3-drug ART for PMTCT	2248	11.7
ART for life	774	4.0
**Antenatal CD4 count above 350 cells/mm** ^**3**^ **(N, %)**		
Yes	11129	57.9
No	7010	36.5
Unknown	1080	5.6
**Antenatal CD4 count (median, IQR) (N** = **18139**)		
	405	280–563
**Antenatal viral load above 5 log**_****10****_ **copies/mL (N, %**)		
Yes	2097	10.9
No	14037	73.0
Unknown	3085	16.1
**Antenatal viral load (log**_**10**_ **copies/mL) (median, IQR) (N** = **16134**)		
	4.0	3.3–4.6

Abbreviations: ART, antiretroviral therapy; ARV, antiretroviral; IQR, interquartile range; PMTCT, prevent mother-to-child transmission.

^a^Information on the date of birth was not available in HIVNET024 and ZEBS. As the enrollment into these studies took place in 2001–2003 and 2001–2004, respectively, these children were classified into the 2000–2004 category.

### HEU Child Mortality

**Figure 2.**
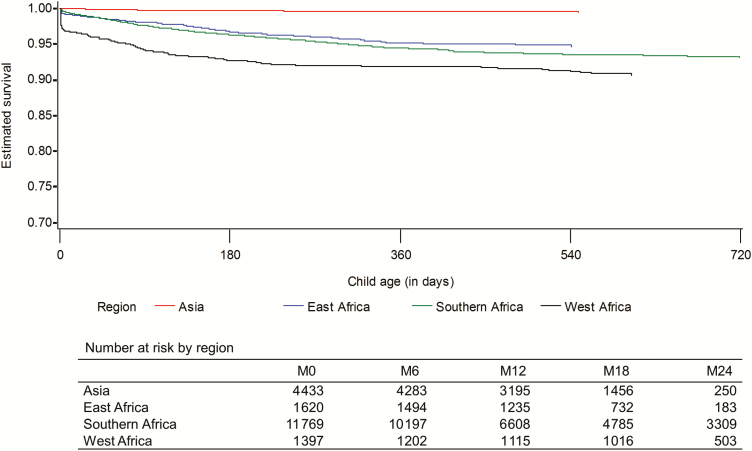
Kaplan-Meier estimates of 24-month survival from birth by geographical region.

Cumulative incidence of death was 2.1% (394/18012; 95% CI, 1.9–2.3), 3.1% (575/17176; 95% CI, 2.9–3.4), 4.5% (797/12153; 95% CI, 4.2–4.8), and 5.5% (884/4245; 95% CI, 5.1–5.9) by age 3, 6, 12, and 24 months, respectively. Median age at death was 111 days (IQR, 37–244). Mortality varied from 0% in PHPT-5 1st [42] to 17.8% in Ditrame-ANRSb [26] (Table 1). Stratified by geographical region (Figure 2), 24-month survival probability was significantly higher in Asia than in Africa (*P* < .0001). Of the 300 children whose mothers died, 17% (n = 51) did not survive after mother’s death. Child mortality declined with increasing age at the time of mother’s death: 52% if mother died within 1 month of delivery, 36% if she died between 1 and 3 months, 20% between 3 and 6 months, 6% between 6 and 12 months, and 4% between 12 and 24 months. Mortality was highest among children with mothers not being on any antiretrovirals (6.1/100 child-years), with mortality in single/dual antiretrovirals for PMTCT, 3.1/100; 3-drug ART for PMTCT, 2.7/100; and ART for life, 3.4/100 child-years. Of note, one- third of the single/dual antiretrovirals for PMTCT and the 3-drug ART for PMTCT groups, respectively, were comprised of mothers of children in PHPT trials where child deaths were rarely observed, which might explain lower mortality rates in these 2 groups.

### Association With Maternal/Child Characteristics

Univariably, LBW children were at 3-fold risk of dying as were never-breastfed children (Table 3). Children whose mother had died were 16 times as likely to die compared to children whose mothers survived; maternal antiretroviral exposure was associated with reduced child mortality, but this did not reach statistical significance. Adjusting for region, maternal antenatal CD4 count, maternal antiretroviral exposure, and maternal vital status, LBW and never breastfeeding remained significantly associated with increased mortality (Table 3). The association between maternal ART for life and reduced child mortality became statistically significant (adjusted hazard ratio [aHR], 0.5; 95% CI, 0.3–0.9) after adjusting for maternal CD4 count (HR, 0.72 in univariate analysis, declining to 0.54 after adjusting for maternal CD4 count only). Associations between mortality and the other antiretroviral categories did not reach statistical significance (single/dual antiretrovirals: aHR, 0.78; 95% CI, 0.50–1.22 and 3-drug ART for PMTCT: aHR, 0.66; 95% CI, 0.39–1.13). Children whose mother had died remained at a substantially increased risk of death (aHR, 11.1).

**Table 3.** Factors Associated With Mortality Through 24 Months of Age in Human Immunodeficiency Virus–Exposed Uninfected Children (n = 19219)

Factor	Total Number of Children (n = 19219)	Total Number of Deaths (n = 884)	Mortality Rate per 100 Child-Years of Follow-up	Univariate Model				Multivariable Model			
				Hazard Ratio	95% CI		*P* Value	Adjusted Hazard Ratio	95% CI		*P* Value
**Region**							<.0001				<.0001
Africa	14786	862	4.6	Ref.				Ref.			
Asia	4433	22	0.4	0.08	0.04	0.17		0.05	0.02	0.11	
**Residence**							0.22				
Rural	1868	101	3.5	Ref.							
Urban/Peri-urban	17351	783	3.7	0.84	0.64	1.11					
**Sex** ^**a**^							0.28				
Male	9839	467	3.8	Ref.							
Female	9376	417	3.6	0.93	0.82	1.06					
**Birth weight**							<.0001				<.0001
≥2500 g	16553	613	2.9	Ref.				Ref.			
<2500 g	2321	246	9.1	3.09	2.66	3.58		2.92	2.51	3.39	
Unknown	245	25	8.1	2.21	1.47	3.33		2.40	1.60	3.61	
**Breastfeeding**							<.0001				<.0001
Ever breastfed	13418	700	4.2	Ref.				Ref.			
Never breastfed	5801	184	2.5	2.76	2.14	3.57		2.48	1.95	3.16	
**Maternal age at delivery (years**)							0.42				
15–20	2074	103	4.4	Ref.							
21–25	6159	276	3.9	0.91	0.73	1.15					
26–30	5175	231	3.8	0.91	0.72	1.16					
31–35	2545	99	3.2	0.80	0.61	1.06					
>35	1011	35	2.8	0.73	0.50	1.08					
Unknown	2255	140	3.5	1.25	0.43	3.68					
**Maternal education**							0.03				
None/Primary	8676	389	3.7	Ref.							
Secondary or above	9688	477	3.7	0.88	0.76	1.03					
Unknown	855	18	3.3	0.23	0.05	1.05					
**Maternal CD4 count (cells/ mm** ^**3**^)							<.0001				<.0001
≥350	11129	417	3.0	Ref.				Ref.			
<350	7010	403	4.6	1.58	1.38	1.82		1.43	1.24	1.66	
Unknown	1080	64	5.6	1.55	1.16	2.07		1.49	1.12	1,99	
**Maternal antiretroviral category**							0.28				0.13
No ARVs	4392	305	6.0	Ref.				Ref.			
Single or double ARV for PMTCT	11805	469	3.1	0.67	0.42	1.07		0.78	0.50	1.22	
3-drug ART for PMTCT	2248	73	2.7	0.61	0.34	1.07		0.66	0.39	1.13	
ART for life	774	37	3.4	0.72	0.38	1.37		0.51	0.28	0.94	
**Maternal vital status (time-dependent**)							<.0001				<.0001
Alive	^b^	833	3.5	Ref.				Ref.			
Dead		51	35.7	15.9	11.9	21.2		11.08	8.25	14.89	

Variable trial/study was included in all models as random effects.

Abbreviations: ART, antiretroviral therapy; ARV, antiretroviral; CI, confidence interval; HR, hazard ratio; PMTCT, prevent mother-to-child transmission.

^a^Excluding n = 4 with no information on sex.

^b^Not applicable for time-dependent variables.

Additional analyses, including ever-breastfed children only (n = 13418) and treating breastfeeding cessation as a time-dependent variable, showed mortality risk to be significantly increased after breastfeeding cessation. Adjusting for region, birth weight, maternal CD4 count, and maternal antiretroviral exposure, breastfeeding cessation was associated with a 12.5-fold (95% CI, 10.3–15.3) risk of death. In this model, children whose mothers received 3-drug ART (both PMTCT and for life) were at significantly lower risk of death (aHR, 0.51; 95% CI, 0.30–0.85 for 3-drug ART for PMTCT and aHR, 0.45; 95% CI, 0.22–0.92 for ART for life) than children whose mothers did not receive antiretrovirals (ARVs).

### Sensitivity Analyses

To investigate the sensitivity of our assumption on 44 women with no information on ARV exposure, we ran the analyses and excluded these women; the aHRs were virtually unchanged. Further, 2 additional analyses were carried out to verify the effects of the inclusion of 1032 children with no information on weaning date and our assumption on their breastfeeding cessation at 6 months. After excluding these children, in the model with breastfeeding treated as a fixed effect, all aHRs were comparable to results shown in Table 3 (aHR, 3.0; 95% CI, 2.3–3.9 vs aHR, 2.5; 95% CI, 2.0–3.2). When breastfeeding was treated as a time-dependent variable, the risk related to breastfeeding cessation increased slightly but remained comparable to results presented in Table 3 (aHR, 16.9; 95% CI, 13.5–21.1 vs aHR, 13.1; 95% CI, 10.7–16.0).

### Combined Effects of Maternal ART and Breastfeeding

Mortality by age 24 months differed significantly by breastfeeding and maternal 3-drug ART status at a given time (*P* < .0001; Table 4). Compared to not currently breastfed children with mothers not receiving 3-drug ART (category A in Table 4), mortality risk in not currently breastfed children with mothers receiving 3-drug ART (category B) was significantly reduced (HR, 0.6). In the absence of maternal 3-drug ART, currently breastfed children (category C) were significantly less likely to die (HR, 0.07). Currently breastfed children whose mothers were receiving 3-drug ART (category D) had the lowest mortality risk (HR, 0.04).

**Table 4.** Multivariate Analysis on the Effects of Breastfeeding and Maternal 3-Drug Antiretroviral Therapy on Child Mortality (n = 24186 child-years)

Category	Child Currently Breastfed^a^	Mother Being on 3-Drug Antiretroviral Therapy^a^	Number of Child-Years	Number of Deaths	Adjusted Hazard Ratio^b^	95% Confidence Interval		*P* Value
								<.0001
**A**	No	No	14119	552	Ref.			
**B**	No	Yes	1027	44	0.63	0.45	0.87	
**C**	Yes	No	7874	269	0.08	0.06	0.09	
**D**	Yes	Yes	1166	19	0.04	0.03	0.07	

^a^Time-dependent variables.

^b^Variable trial/study was included as random effects. Also adjusted for region, maternal antenatal CD4 count, and birth weight.

### Adjusted Attributable Fractions of Risks

To investigate the impact of LBW, never breastfeeding, mother not on 3-drug ART for life, and maternal death, we estimated the aAFs of risks based on the parameter estimates obtained from our final model (Table 3). Mother not receiving 3-drug ART for life accounted for 45.6% (95% CI, 19.1–63.9) of child deaths by age 24 months. LBW accounted for an estimated 16.2% of child deaths by age 24 months, never breastfeeding for 10.8%, and maternal death for 4.3%. Combined, these 4 factors explained 63.6% (95% CI, 45.7–76.6) of deaths by age 24 months. The aAFs of risks at 6 and 12 months related to these 4 factors did not significantly differ from those at 24 months (Table 5).

**Table 5.** Estimated Adjusted Attributable Fractions and 95% Confidence Intervals of Risk Factors for Mortality in Human Immunodeficiency Virus–Exposed Uninfected Children at Different Time Points

Expected Number of Deaths Given the Distribution at 6, 12, and 24 Months	0–6 Months			0–12 Months			0–24 Months		
	755			1054			1444		
	Number of Deaths Attributable to the Risk Factor(s)	aAF (%)	95% CI	Number of Deaths Attributable to the Risk Factor(s)	aAF (%)	95% CI	Number of Deaths Attributable to the Risk Factor(s)	aAF (%)	95% CI
Mother not receiving 3-drug antiretroviral therapy for life	342	47.9	20.0–65.8	466	46.9	15.6–64.9	620	45.6	19.1–63.9
Low birth weight	140	18.5	15.1–21.9	184	17.4	14.2–20.6	234	16.2	13.1–19.2
Breastfeeding never initiated	88	11.7	5.2–19.6	119	11.3	5.0–18.9	157	10.8	4.9–17.9
Mother not being alive	44	5.9	3.1–13.5	53	5.1	2.7–11.5	62	4.3	2.3–9.3
All 4 factors above	486	66.2	48.6–78.4	666	65.0	47.2–77.7	891	63.6	45.7–76.6

Abbreviations: aAF; adjusted attributable fraction; CI, confidence interval.

## DISCUSSION

Using data from 21 studies/trials undertaken between 1995 and 2015 in Africa and Asia, our findings suggest that where mothers are alive, on ART for life, and breastfeed their infants, 24-month mortality in HEU children is substantially reduced.

As reported previously [50–52], LBW, prevalent in 12% of these HEU children, was a major risk factor for mortality. However, the negative consequences of LBW and non-breastfeeding may be even greater in settings outside the context of well-resourced research studies. Almost half of HEU deaths occurred in the first 3 months of life and two-thirds before age 6 months, highlighting the importance of intervening programmatically in this early period.

The survival of mothers living with HIV had a major effect on the survival of HEU children; this association has also been reported among HUU children [53]. In our analyses, the death of a mother shortly after delivery was most hazardous for the survival of her HEU child.

Our results suggest that the risk of mortality in HEU children is reduced when mothers are on ART either until breastfeeding cessation or for life. Mother’s initiation and continuation of ART likely improves her own health, which in turn increases the chances of child survival through better breastfeeding practices, reduced exposure to comorbidities, improved mother’s care capacity, and other unmeasured benefits at the household level.

The estimated AFs differed from the aHRs. This indicates that the impact at the population level, which reflects prevalence of risk factors, differs from that at the individual level. The CI around the AF estimate of mother not receiving 3-drug ART for life was particularly wide, and caution is required in interpreting this result. Our estimated AFs show that 36% of HEU child mortality at age 24 months could not be accounted for by the 4 risk factors identified and highlight that HEU children are also at risk of death from other common causes of child mortality. The lack of contemporaneous mortality data from HUU children meant that we were unable to categorically comment whether HEU children are at any greater mortality risk if these 4 risk factors are fully addressed.

Although the analyses include a large number of HEU children from diverse settings, interpretation of findings is hindered by lack of detail on potentially important variables including gestational age, neonatal care practices (early initiation and type of breastfeeding), and household exposure to opportunistic infections (such as tuberculosis). These factors may account for much of the remaining 36% mortality.

Relatively few women were on ART for life, and studies generally followed earlier WHO guidelines on HIV and infant feeding, recommending breastfeeding for about 6 months only. Although we confirm the associations between reduced mortality risk and maternal ART for life and breastfeeding, our data does not allow us to fully capture the complex associations between different factors that influence a child’s survival outcome. The women on ART in our study are a highly select population, and we cannot comment on the extent to which such reductions are facilitated by study-specific factors and whether such associations would be equally observed in women on ART in standard-of-care settings. Further, due to lack of data, we were unable to allow for cotrimoxazole prophylaxis and childhood immunization, which are used to prevent infectious morbidity in young children.

We were also unable to differentiate small-for-gestational age from premature infants in those with LBW. About 11% of all infants in Eastern and Southern Africa are born with LBW; in Southeast Asia it is around 28% [54]. LBW has been associated with HIV infection in pregnant women in sub-Saharan Africa and with the protease inhibitor class of ART during pregnancy [55–57]. While the primary drivers for LBW may vary by region and HIV exposure, the relationship between LBW and mortality in both HEU and HUU children is clear and strong and has immediate programmatic implications.

The combination of 3-drug ART was introduced in about the middle of the timespan covered by the studies included; ART eligibility criteria varied over time as did inclusion for trials. Although we could not allow for these trends, these factors could have introduced selection bias. Each study had its own criteria for eligibility, including CD4 counts. Data included in our analyses were heterogeneous in terms of feeding and duration of follow-up, which limits our ability to generalize the results beyond these studies. Finally, exclusion of 457 children whose breastfeeding status was unknown might have led to underestimation of mortality rates as almost 29% (132/457) of these children died before age 12 months; 105 died before age 1 month, 17 between age 1 and 3 months, and 10 died thereafter.

We show substantial regional differences in 24-month mortality, which deserves further investigation, as do the issues surrounding breastfeeding. Explaining the missing fraction of HIV-related and other external mortality risk factors requires prospectively collected data from both HEU and HUU populations. It remains unclear whether HEU infants and children

are immunologically impaired at a clinically significant level or whether increased exposure to opportunistic infections because of living in HIV-affected households would explain the missing fraction. Perhaps the most germane question is whether increasing rollout of lifelong ART among women living with HIV and fully supporting optimal infant feeding practices will mitigate the patterns of risks identified in these historical cohorts. With more and more women living with HIV being initiated on ART, understanding the interactions between fetal HIV and ART exposure, the effects of prematurity or small-for-gestational age on mortality, and the effects of other long-term outcomes including early child development, infectious morbidity, and the risk of noncommunicable diseases will be increasingly important.

## CONCLUSIONS

Our findings show that not-breastfeeding and LBW were associated with considerable mortality risk and suggest that maternal ART, initiated before or during pregnancy, may substantially reduce child mortality in the first 2 years of life. With increasing numbers of HIV-infected pregnant women being initiated on ART, this would provide hope for reducing overall child mortality in settings of high HIV prevalence. The importance of delivering effective integrated care so that women living with HIV are not only initiated on ART but are also linked with other essential elements of maternal and child healthcare is clear. Eliminating pediatric HIV and improving the survival, health, and development of HEU children should not be separate from improving the well-being of mothers and children not affected by HIV, and our metric of success needs to evolve to “HIV-free survival and development.” While integrated programs and coordinated research and monitoring are unquestionably possible, continued global investment in these responses is perhaps the greatest challenge.

## Supplementary Data

Supplementary materials are available at *Clinical Infectious Diseases* online. Consisting of data provided by the authors to benefit the reader, the posted materials are not copyedited and are the sole responsibility of the authors, so questions or comments should be addressed to the corresponding author.

Supplementary Files

## Notes

***Author contributions.*** R. B., M. L. N., and N. R. initiated and set the objectives of the collaboration, defined the statistical analysis plans, and substantially contributed to the writing of the manuscript. S. A. undertook the literature review, managed the data pooling, participated in the definition of the statistical analysis plan, performed statistical analysis, and wrote the first draft of the paper. M. R. and P. J. substantially contributed to the statistical analysis. G. J., J. H., T. F., G. G., L. K., R. S., V. L., S. L., R. C. B., T. D., and S. L. C. critically reviewed the manuscript and substantially contributed to the interpretation of the results. All other coauthors reviewed the manuscript.

***Acknowledgments.*** BAN: Charles van der Horst, Denise Jamieson; Ditrame-ANRSa: Christiane Welffens-Ekra, Philippe Msellati; Ditrame-ANRSb: Nicolas Meda, Philippe Van de Perre; Ditrame Plus: Marguerite Timité-Konan, Clarisse Bosse; Good Start: Mickey Chopra, Debra Jackson, Vundli Ramokolo, Ameena Goga; HIVIGLOB: J. Brooks Jackson, Laura A. Guay, Philippa Musoke, Mary Glenn Fowler, Michael C. Mubiru; PEP: Mike Urban (University of Stellenbosch); PHPT: Marc Lallemant, principal investigator of the PHPT clinical trials, all PHPT coinvestigators, the PHPT staff who helped collect and manage the data, and Nicolas Salvadori for the preparation of the data-set of the PHPT studies. PROMOTE2: Diane Havlir, Theodore Ruel. SWEN: Amita Gupta, Jayagowri Sasty, Harjot K. Singh; ZEBS: The late Moses Sinkala (Lusaka District Health Management Team), Chipepo Kankasa (University Teaching Hospital), Donald M. Thea (Boston University), Grace M. Aldrovandi (University of California–Los Angeles).

***Funding.*** This work was funded by the WHO.

***Potential conflicts of interest.*** All authors: No reported conflicts of interest. All authors have submitted the ICMJE Form for Disclosure of Potential Conflicts of Interest. Conflicts that the editors consider relevant to the content of the manuscript have been disclosed.
