# Supplementary material for: Contribution of Maternal Antiretroviral Therapy and Breastfeeding to 24-Month Survival in Human Immunodeficiency Virus-Exposed Uninfected Children: An Individual Pooled Analysis of African and Asian Studies
Source: Clin Infect Dis. 2017 Dec 21;66(11):1668–77. doi: 10.1093/cid/cix1102 (PMC5961296; doi:10.1093/cid/cix1102)
Supplement: Supplementary Files [file cix1102_suppl_supplementary_files.docx]

**SUPPLEMENTARY FILE**

**Table 1: Characteristics of included studies**

|  | **Study/Trial** | **Country** | **Enrolment** | **Study design** |
| --- | --- | --- | --- | --- |
|  |  |  |  |  |
| 1 | BAN | Malawi | 2004-2010 | RCT* |
| 2 | [Ditrame (ANRSa)](file:///C:\Users\SHINO\Dropbox\HEU%20pooled\HEU%20description%20(Enregistré%20automatiquement).xlsx#RANGE!_ENREF_26) | Côte d'Ivoire, Burkina Faso | 1995-1998 | RCT |
| 3 | [Ditrame (ANRSb)](file:///C:\Users\SHINO\Dropbox\HEU%20pooled\HEU%20description%20(Enregistré%20automatiquement).xlsx#RANGE!_ENREF_26) | Côte d'Ivoire, Burkina Faso | 1996-1997 | RCT |
| 4 | [Ditrame Plus](file:///C:\Users\SHINO\Dropbox\HEU%20pooled\HEU%20description%20(Enregistré%20automatiquement).xlsx#RANGE!_ENREF_26) | Côte d'Ivoire | 2001-2003 | Observational study |
| 5 | Good Start | South Africa | 2002-2004 | Observational study |
| 6 | HIVIGLOB/SWEN Uganda | Uganda | 2004-2006 | RCT |
| 7 | HIVNET 024 | Malawi, Tanzania, Zambia | 2001-2003 | RCT |
| 8 | Kesho Bora | Burkina-Faso, Kenya,  South Africa | 2005-2008 | RCT |
| 9 | Mashi | Botswana | 2001-2003 | RCT |
| 10 | Mma Bana | Botswana | 2006-2008 | RCT |
| 11 | PEP | South Africa | 2001-2002 | RCT |
| 12 | PHPT 1 | Thailand | 1997-1999 | RCT |
| 13 | PHPT 2 | Thailand | 2000-2003 | RCT |
| 14 | PHPT5-1st | Thailand | 2009-2010 | RCT |
| 15 | PHPT5-2nd | Thailand | 2010-2012 | RCT |
| 16 | PROMOTE2 | Uganda | 2009-2012 | RCT |
| 17 | SWEN | India | 2002-2007 | RCT |
| 18 | Tshipidi | Botswana | 2010-2012 | Observational study |
| 19 | [Vertical Transmission Study](file:///C:\Users\SHINO\Dropbox\HEU%20pooled\HEU%20description%20(Enregistré%20automatiquement).xlsx#RANGE!_ENREF_28) | South Africa | 2001-2005 | Observational study |
| 20 | ZEBS | Zambia | 2001-2004 | RCT |
| 21 | ZVITAMBO | Zimbabwe | 1997-2000 | RCT |

*RCT: Randomized controlled trial

**Table 2: Distribution of mothers according to ARV exposure category (%)**

| **Trial/study** | **No ARVs** | **Single/ double ARV for PMTCT** | **3-drug ART for PMTCT** | **ART for life** |
| --- | --- | --- | --- | --- |
| BAN | 0 | 64.3 | 35.7 | 0 |
| Ditrame (ANRSa) | 48.1 | 51.9 | 0 | 0 |
| Ditrame (ANRSb) | 100 | 0 | 0 | 0 |
| Ditrame Plus | 0 | 98.1 | 1.9 | 0 |
| Good Start | 20.5 | 79.5 | 0 | 0 |
| HIVIGLOB/SWEN Uganda | 0 | 100 | 0 | 0 |
| HIVNET024 Malawi | 2.5 | 97.5 | 0 | 0 |
| HIVNET024 Tanzania | 1.42 | 98.6 | 0 | 0 |
| HIVNET024 Zambia | 3.7 | 96.3 | 0 | 0 |
| Kesho Bora Burkina Faso | 0 | 47.9 | 37.8 | 14.3 |
| Kesho Bora Kenya | 0 | 52.9 | 31.8 | 15.4 |
| Kesho Bora South Africa | 0 | 47.8 | 52.2 | 0 |
| Mashi | 0 | 93.7 | 1.8 | 4.5 |
| Mma Bana | 0 | 0 | 70.9 | 29.1 |
| PEP | 100 | 0 | 0 | 0 |
| PHPT-1 | 0 | 100 | 0 | 0 |
| PHPT-2 | 0 | 100 | 0 | 0 |
| PHPT5-1st | 0 | 66.3 | 33.7 | 0 |
| PHPT5-2nd | 0 | 0 | 100 | 0 |
| PROMOTE2 | 0 | 0 |  | 100 |
| SWEN | 29.1 | 67.7 | 3.2 | 0 |
| Tshipidi | 0 | 67.1 | 20.3 | 12.6 |
| VTS | 0 | 100 | 0 | 0 |
| ZEBS | 0 | 99.2 | 0.8 | 0 |
| Zvitambo | 100 | 0 | 0 | 0 |
| **Total** | **22.9** | **61.2** | **11.8** | **4.1** |
